# Electronic Event–based Surveillance for Monitoring Dengue, Latin America

**DOI:** 10.3201/eid1807.120055

**Published:** 2012-07

**Authors:** Anne G. Hoen, Mikaela Keller, Aman D. Verma, David L. Buckeridge, John S. Brownstein

**Affiliations:** Children’s Hospital Boston, Boston, Massachusetts, USA (A.G. Hoen, J.S. Brownstein);; Harvard Medical School, Boston (A.G. Hoen, J.S. Brownstein);; Institut de Recherche en Informatique et Automatique, Lille, France (M. Keller);; Université de Lille, Lille (M. Keller);; McGill University, Montreal, Quebec, Canada (A.G. Hoen, A.D. Verma, D.L. Buckeridge, J.S. Brownstein);; and Agence de la Santé et des Services Sociaux de Montreal, Montreal (D.L. Buckeridge)

**Keywords:** dengue, viruses, communicable diseases, disease outbreaks, internet, population surveillance, electronic event–based surveillance, public health

## Abstract

The current dengue epidemic in Latin America represents a major threat to health. However, surveillance of affected regions lacks timeliness and precision. We investigated real-time electronic sources for monitoring spread of dengue into new regions. This approach could provide timely estimates of changes in distribution of dengue, a critical component of prevention and control efforts.

## Abstract 2

Dengue, a potentially fatal disease, is spreading around the world. An estimated 2.5 billion people in tropical and subtropical regions are at risk. Early detection of outbreaks is crucial to prevention and control of dengue virus and other viruses. Case reporting may often take weeks or months. Therefore, researchers explored whether electronic sources of real-time information (such as Internet news outlets, health expert mailing lists, social media sites, and queries to online search engines) might be faster, and they were. Although information from unofficial sources should be interpreted with caution, when used in conjunction with traditional case reporting, real-time electronic surveillance can help public health authorities allocate resources in time to avert full-blown epidemics.

Dengue, a potentially fatal viral disease, has been recognized for >200 years (*1*). Once sporadic and limited geographically, dengue viruses (DENVs) and their mosquito vectors have spread globally, putting an estimated 2.5 billion persons at risk throughout the tropical and subtropical regions of the world (*2*). Factors contributing to the dramatic expansion of DENV activity include demographic changes such as population growth, urbanization, and globalization, and reductions in vector control and other public health measures (*3*). Because of the nature of passive surveillance, conventional systems have limited ability in identifying new epidemics quickly (*1*), thus suggesting a role for alternative information sources.

Free or low-cost sources of unstructured information, such as Internet news outlets, health expert mailing lists, social media sites, and queries to online search engines, when computationally filtered and mined, can provide detailed local and near real-time data on potential or confirmed disease outbreaks (*4*). For dengue in particular, our group and others recently reported on a set of Google search terms that parallel temporal trends in official dengue case counts (*5**,**6*). These event-based data sources can provide insight into new and ongoing public health challenges in areas of the world with limited public health reporting infrastructure.

Few studies have investigated the value of unofficial sources for monitoring recent geographic expansion of infectious disease risk. Using dengue as a case study, we report on the utility of electronic outbreak surveillance for real-time monitoring of recent infectious disease spread.

## The Study

We focused on the geographic range of DENVs in Latin America and the Caribbean, where dengue is widespread and expanding in range. We attempted to identify areas contiguous with previously known dengue-endemic zones where new DENV transmission is occurring by using reports of recent outbreaks.

Known dengue-endemic areas were defined as dengue risk areas identified by the US Centers for Disease Control and Prevention (Atlanta, GA, USA) Health Information for International Travel (commonly referred to as the Yellow Book), 2010 (*7*) and 2012 (*8*) editions. Each edition of this book reflects the known distribution of dengue risk in the prior 2 years. To characterize spread according to the Yellow Book, we identified areas that were classified as no known dengue risk in 2010 but were changed to risk areas in the 2012 edition (hereafter referred to as new dengue-endemic areas).

Outbreak data for December 1, 2009–March 18, 2011, were collected from HealthMap (http://www.healthmap.org/en/an), an open access online infectious disease outbreak monitoring system (*9**,**10*). HealthMap integrates outbreak-related data from >30,000 electronic sources, including the news media, ProMED-mail, and other electronic public health reporting sources, by using algorithms to classify the diseases and locations associated with each report. Because we wanted to identify spread into new dengue-endemic zones, we limited our analyses to areas that were identified as having no known dengue risk in the 2010 Yellow Book but that were contiguous with >1 risk areas in the 2010 Yellow Book. We identified 53 dengue outbreaks distributed in 60 of these areas.

**Figure 1:**
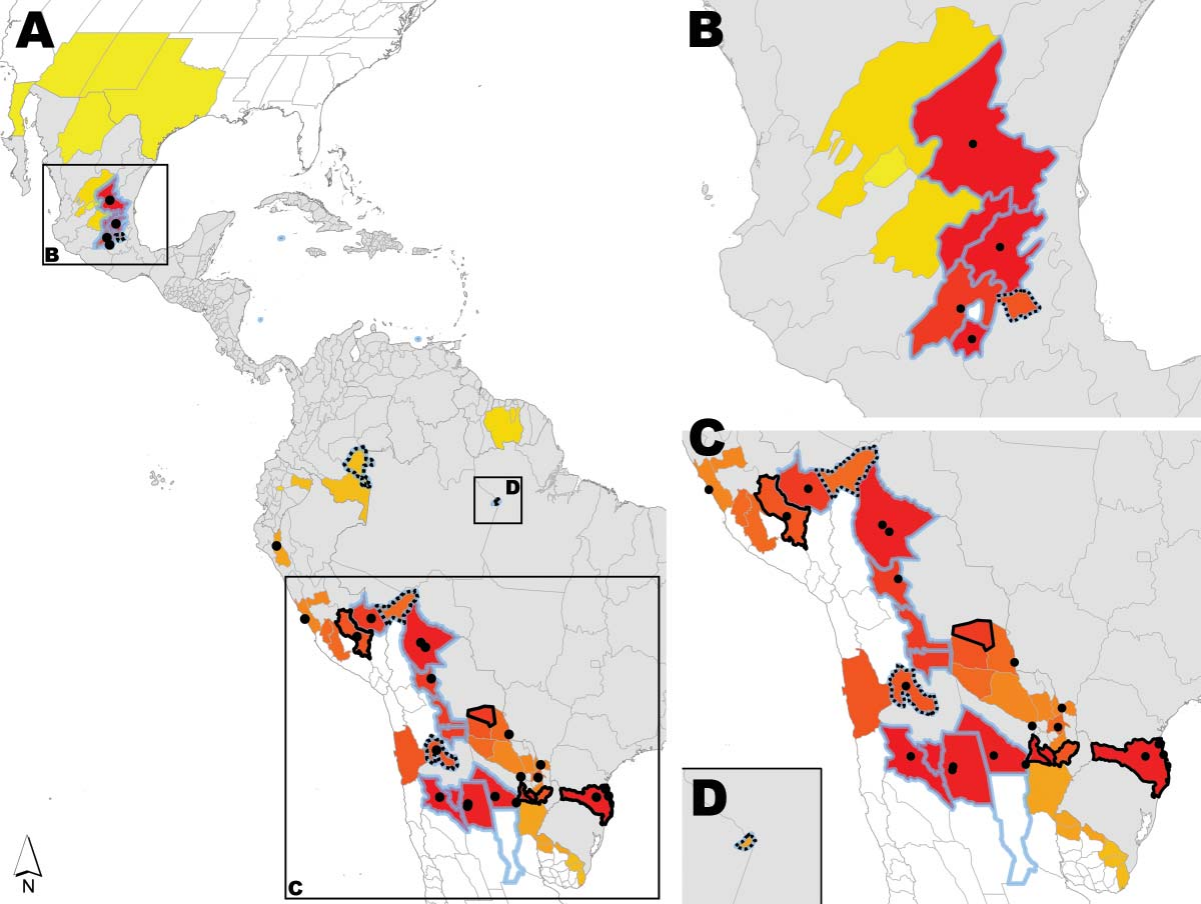
A) Regions in Latin America analyzed for dengue. B) Central Mexico; C) central South America; D) eastern Amazonas, Brazil. Thin gray lines indicate boundaries of first-level administrative units. Areas to which dengue was identified in the 2010 Yellow Book are shaded in gray. New dengue-endemic areas identified in the 2012 Yellow Book are outlined in blue. Dots indicate HealthMap dengue-related alerts. Modeled HealthMap alert probability density surface is shown in a gradient from yellow to red with yellow areas predicted as having lower alert densities and red areas predicted as having higher alert densities according to the model. Areas outlined with heavy black solid lines were classified as high HealthMap alert density but were not identified in either Yellow Book edition as dengue risk areas. Areas outlined with heavy black dashed lines were classified as low HealthMap alert density but were identified in the 2012 Yellow Book as areas at risk for dengue.

We fitted a bivariate Gaussian mixture model to the extracted HealthMap alerts to model a continuous surface of outbreak density (Technical Appendix). This modeled outbreak probability density surface represents a risk map of recent DENV spread into areas of previously unknown dengue endemicity according to the 2010 Yellow Book (Figure 1). We compared our map with the geographic distribution of new dengue-endemic areas identified in the 2012 Yellow Book. Details of the datasets, models, and statistical methods are available in the Technical Appendix.

**Figure 2:**
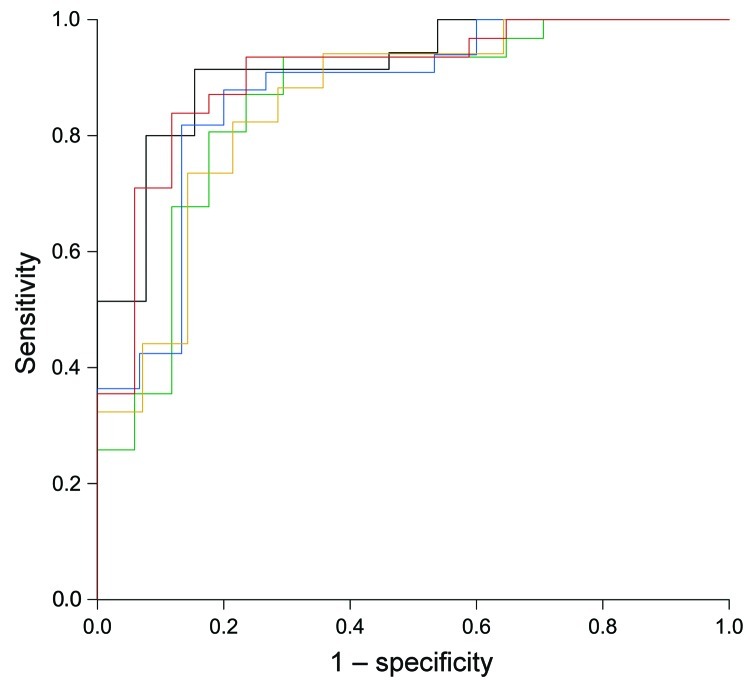
Receiver-operating characteristic plot of 5-fold cross-validated HealthMap alert density-based classification with new dengue-endemic areas identified by the 2012 Yellow Book as the standard.

Figure 1 shows that high dengue outbreak activity occurred adjacent to previously recognized dengue-endemic zones in 6 states in central Mexico and in parts of northern Argentina, southern Brazil, Bolivia, and Paraguay. We used receiver-operating characteristic analysis with cross-validation (Figure 2) to set a threshold dengue report density that best identifies new dengue-endemic areas (Figure 1; Technical Appendix). Of the 19 new dengue-endemic areas reported in the 2012 Yellow Book, this threshold identified 14 (74%) as being at elevated risk of endemicity, according to the dengue outbreak probability density estimated by our model. Of the 41 areas that remained unidentified as dengue-endemic areas in the 2012 Yellow Book, our model classified 35 (85%) as having reduced risk of endemicity.

When compared with the Yellow Book, our model incorrectly classified 6 areas as at elevated risk (Figure 1). All alerts in these areas described outbreaks of cases acquired in a nearby known dengue-endemic region of the country. One alert also warned of the recent discovery of dengue vector mosquito larvae by the local surveillance program. The model also classified 5 Yellow Book dengue-endemic areas as at reduced risk (Figure 1). Four of these areas were rural or isolated. Although other explanations likely exist, the low observed sensitivity in these areas illustrates certain limits of any system that relies on Internet-based information flow for monitoring disease spread.

## Conclusions

Electronic event–based surveillance systems such as HealthMap and others are frequently used by public health authorities, travelers, physicians and patients, to gain a real-time understanding of global outbreak activity. The HealthMap dengue feed, DengueMap, is currently part of the online dengue information resource of the Centers for Disease Control and Prevention (http://www.cdc.gov/dengue/). Used in combination with traditional case reporting, HealthMap and other electronic surveillance systems have proven value for enhancing the timeliness of outbreak discovery and information dissemination (*11*). However, these information sources may also provide added value for monitoring ongoing spread.

Although the signal of DENV activity detected by HealthMap is relatively robust, it has certain limitations. First, the signal tends to be sparse in areas with limited reporting because of low population density or incomplete coverage by the news or social media. Second, the signal can be surrounded by background noise because separating reports caused by cases in travelers from true autochthonous transmission is difficult with automated methods. By limiting our analysis to areas contiguous with known dengue-endemic areas and smoothing outbreak alerts into an outbreak-density surface, we were able to identify a reliable signal of dengue spread. Although this analysis was performed retrospectively, the timeliness of this signal far outperforms any traditional surveillance data stream. Passive case report-based surveillance systems typically operate at a delay of weeks to months, which limits their value for providing a picture of geographic spread, especially on an international scale where surveillance delays may be even more prolonged.

We have demonstrated a novel approach to real-time monitoring of recent expansion of DENV activity in Latin America. Using outbreak reports captured by HealthMap, we identified a signal of geographic expansion of dengue activity that would precede official reports of the geographic distribution of dengue-endemic areas. Currently, no reliable surveillance system is in widespread use that reports the distribution of DENV activity on an ongoing basis and enables near real-time monitoring of trends in geographic expansion. Such a system should enhance the ability of regional and global public health authorities to dynamically allocate resources within a time frame that might effectively avert a full-blown epidemic. Like other large-scale surveillance data sources, our results must be interpreted cautiously. However, when used in conjunction with traditional surveillance methods, our approach has the potential to provide a timely estimate of changes in the geographic distribution of dengue, a critical component of targeted prevention and control efforts.

## Supplementary Material

Technical Appendix Bivariate Gaussian mixture model applied to extracted HealthMap alerts to model a continuous surface of outbreak density.
