# Supplementary material for: Electronic Event–based Surveillance for Monitoring Dengue, Latin America
Source: Emerg Infect Dis. 2012 Jul;18(7):1147–50. doi: 10.3201/eid1807.120055 (PMC3376807; doi:10.3201/eid1807.120055)
Supplement: Technical Appendix — Bivariate Gaussian mixture model applied to extracted HealthMap alerts to model a continuous surface of outbreak density. [file 12-0055-Techapp_3p.pdf]

Article DOI: http://dx.doi.org/10.3201/eid1808.120055

Electronic Event–based Surveillance for Monitoring Dengue, Latin America

Technical Appendix

Bivariate Gaussian mixture model applied to extracted HealthMap alerts to model a continuous surface of outbreak density

Datasets

CDC Yellow Book

We obtained the geographic boundaries of dengue risk areas reported in the U.S. Centers for Disease Control and Prevention's Health Information for International Travel (commonly referred to as the Yellow Book), 2010 and 2012 editions (*1*,*2*). The Yellow Book is published every 2 years as a reference for health care providers who advise international travelers on health risks. The Yellow Book classifies regions of the world into dengue risk areas and areas with no known dengue risk on the basis of whether dengue is considered to be endemic in these areas. This classification relies on expert-reviewed reports and peer-reviewed publications, as well as communications with subject-matter authorities. Areas are drawn at the scale of first-level administrative units (subnational regions such as state or province). For our study, we used only areas that had been labeled as no known risk but were adjacent to and contiguous with risk areas in the 2010 Yellow Book.

Because the Yellow Book is intended as information for clinicians and travelers and does not provide detailed information on the precise criteria that are used in making its classifications, we are limited in our ability to make inferences regarding the exact meanings of the 2 classifications. However, to our knowledge, the Yellow Book provides the most geographically comprehensive and most frequently updated dengue risk map currently available, which motivated its use as our reference map for this study.

HealthMap

HealthMap has been described (*3*). In brief, HealthMap is an online infectious disease monitoring system that captures online information about outbreaks with automated hourly scans of >30,000 electronic sources in 9 languages and then uses a text processing algorithm to classify items by disease and geographic location. HealthMap sources include the news media through Google News and other news aggregators; the moderated listserv ProMED Mail; the GeoSentinal Surveillance Network’s database of illness among travelers; and formal reports from the Food and Agriculture Organization and World Health Organization, among others. Approximately 80% of the volume of HealthMap alerts reflects information captured directly from the news media.

We extracted all HealthMap alerts in the Americas that had been geolocated to first-level administrative areas contiguous with 2010 Yellow Book dengue-positive areas. Our analysis was limited to HealthMap alerts issued from December 1, 2009, when HealthMap began curating Spanish and Portuguese language reports, through March 18, 2011, when we extracted our dataset for this analysis.

Spatial Modeling

We fit a bivariate Gaussian mixture model to our dataset of HealthMap alerts. This is a statistical model of a probability density function made up of a weighted sum of Gaussian densities. While a common application of mixture modeling is cluster detection, mixture models are also used for density estimation. In a spatial context, this enables estimation of the underlying and unobservable continuous density of a set of observed points. Our candidate spatial models included the covariates latitude and longitude represented by Mollweide equal area–projected x- and y-coordinates. Therefore, the computed models are weighted sums of component bivariate Gaussian distributions that represent the probability distribution of HealthMap observations in the study area. We fit bivariate Gaussian mixture models with 1, 2, …, 10 components and diagonal or spherical covariance functions and then selected the best fit model according to the Bayesian information criterion. We ensured that the best-fit model did not fall at the minimum or maximum number of components considered. Using the best-fit model, we then estimated the mean probability density for each first-level administrative unit contiguous with 2010 Yellow Book dengue-positive areas. This value that can be thought of as a model-based estimate of the intensity of HealthMap alerts for each subnational area.

Statistical Methods

Of included administrative areas, we identified all that had been changed from an area with no known dengue risk in the 2010 Yellow Book edition to dengue risk area in the 2012 Yellow Book edition. We hypothesized that these were areas into which dengue had spread between editions of the Yellow Book, and that the areas with the strongest HealthMap alert density would correspond to these areas of recent expansion. To test this hypothesis, we plotted receiver operating characteristic curves to evaluate the sensitivity and specificity of a range of threshold HealthMap alert density values for predicting the occurrence of new dengue risk areas in the 2012 Yellow Book and selected optimally predictive density threshold cutoffs by using the Youden statistic (*4*). To avoid over fitting, we performed receiver operating characteristics analysis with 5-fold cross validation.

Mapping was performed by using ArcGIS Desktop version 10 (ESRI, Redlands, CA, USA). Statistical analyses were performed by using statistical software version 2.12.2 with the packages MCLUST and ROCR (http://cran.r-project.org/bin/windows/base/old.2.12.2).

References

1. Centers for Disease Control and Prevention. CDC health information for international travel 2010. Atlanta: The Centers; 2010.

2. Centers for Disease Control and Prevention. CDC health information for international travel 2012. Atlanta: The Centers; 2012.

3. Freifeld CC, Mandl KD, Reis BY, Brownstein JS. HealthMap: global infectious disease monitoring through automated classification and visualization of Internet media reports. J Am Med Inform Assoc. 2008;15:150–7. PubMed http://dx.doi.org/10.1197/jamia.M2544

4. Fluss R, Faraggi D, Reiser B. Estimation of the Youden index and its associated cutoff point. Biom J. 2005;47:458–72. PubMed http://dx.doi.org/10.1002/bimj.200410135
